# Rural and urban disparities in cardiovascular disease-related mortality in the USA over 20 years; have the trends been reversed by COVID-19?

**DOI:** 10.1016/j.ijcrp.2023.200202

**Published:** 2023-08-30

**Authors:** Saisunder S. Chaganty, Dmitry Abramov, Harriette G.C. Van Spall, Renee P. Bullock-Palmer, Vassilios Vassiliou, Phyo Kyaw Myint, Vijay Bang, Ofer Kobo, Mamas A. Mamas

**Affiliations:** a Keele Cardiovascular Research Group, Centre for Prognosis Research, Keele University, Stoke- on-Trent, UK; b International Heart Institute, Loma Linda University, Loma Linda, CA, USA; c Department of Medicine, McMaster University, Population Health Research Institute, Hamilton, Ontario, Canada; d Department of Cardiology, Deborah Heart and Lung Center, Browns Mills, NJ, USA; e Norwich Medical School, University of East Anglia, 2.06 Bob Champion Research and Education Building, And Norfolk and Norwich Hospital, NR4 7TJ, UK; f Aberdeen Cardiovascular and Diabetes Centre, School of Medicine, Medical Sciences & Nutrition, University of Aberdeen, Aberdeen, Scotland, UK; g Oriion Citicare Hospital, Aurangabad, India; h Department of Cardiology, Hillel Yaffe Medical Center, Hadera, Israel

## Abbreviations

AAMR: Age adjusted mortality ratio
AAPC: Average Annual Percentage change
CDC WONDER: Centers for Disease Control and Prevention Wide-Ranging, Online Data for Epidemiologic Research
COVID-19: Coronavirus disease 2019 (SARS-CoV-2)
CVD: Cardiovascular disease
ICD-10: International Classification of Disease Tenth Revision
IHD: Ischemic heart disease
USA: United states of America

## 1 Introduction

Rural-urban disparities in cause-specific mortality in the USA have been reported for a variety of chronic conditions, including substance misuse, suicides, and organ system diseases [[1], [2], [3], [4]]. This has been on the backdrop of the well-documented phenomenon of rural areas characteristically experiencing worse health and higher mortality rates compared to urban areas [1,5,6]. Studies have attributed this to regional socioeconomic deprivation, less access to healthcare, and reduced awareness in rural compared to urban areas [[7], [8], [9]]. For cardiovascular disease, studies have noted the CVD-related mortality rates in the US declined in the first decade of the 21st century, followed by a plateau [[10], [11], [12]].

Over the past few years, the COVID-19 pandemic has disrupted provision of healthcare delivery both in urban and rural areas of the US, along with a demonstrable loss of life expectancy. The pandemic has brought about significant economic, social, and political challenges. However, there remains limited data around whether rural-urban disparities in cardiovascular-disease related mortality in the US have worsened particularly in the context of the COVID-19 pandemic. Furthermore, there is limited data on how temporal mortality trends in rural and urban areas vary when looking at specific CVD sub-types, and whether the COVID-19 pandemic has reversed the gains differentially amongst different CVD from the last two decades.

Our study aims to study the temporal evolution in cardiovascular disease-related mortality in rural and urban areas in the US from Jan 1st, 1999 to Dec 31st, 2020. We subsequently discuss CVD-related excess mortality in urban/rural areas in the context of the first year of the COVID-19 pandemic (2020) relative to the preceding twenty years (1999–2019).

## 2 Methods

### 2.1 Data source

We collected data from the Centers for Disease Control and Prevention Wide-Ranging Online Data for Epidemiologic Research (CDC WONDER) database. From January 1st, 1999 to December 31st, 2020, cardiovascular-related mortality data was collected using death certificates for U.S. residents. Each death certificate contained a single underlying cause of death, up to twenty additional causes and demographic data. Cardiovascular-related mortality was identified using the ICD10 codes presented in Supplemental Table 1 as the underlying cause of death. The inclusion of multiple types of cardiovascular diseases including diabetes, hypertension, cerebrovascular, and ischemic heart disease is consistent with notable similarities and risk overlap within these conditions [13]. Mortality related to cardiovascular-related disease was queried and stratified according to the following demographics: urban-rural designation (2013 US Census classification), sex, race, and age (<55 years of age versus >55 years of age; these were the pre-defined ranges on the CDC database) [14]. The study was exempt from ethical approval as the CDC WONDER database contains anonymized, publicly available data.

### 2.2 Outcomes

Total deaths and age-adjusted mortality rates (AAMR) per 100,000 population were obtained for each year from 1999 to 2020. The population estimates for the denominators of rates were race-, sex-, and age-specific and were obtained annually for the rural-urban population. For age-adjustment, the National Center for Health Statistics adopted the age distribution of the year 2000 population of the USA as the standard population for the purpose of age adjustments. As a result, the AAMR was calculated using the direct standardization method based on the age group weights from the 2000 U S. population.

### 2.3 Statistical analysis

The AAMR presented per 100,000 population number of deaths are presented as absolute values. The changes in mortality are presented as percentage (%). The Joinpoint Regression Program (Version 4.9.1.0), from the National Cancer Institute was used to calculate the average annual percent change (AAPC) in AAMR and is presented as a percentage with 95% confidence interval (CI). The projected AAMR for 2020 was estimated using a sensitivity analysis of the AAPC from the one-decade prior (2010–2019), with statistical significance set at the p < 0.05 value (see Supplementary Table 3). Excess AAMR was estimated by comparing the projected and the actual AAMR for the first year of the pandemic. This allowed the calculation of the proportion of excess AAMR (as a percentage) and estimation of the number of excess deaths.

## 3 Results

### 3.1 Baseline characteristics & overall combined CVD-related mortality

From 1999 to 2019, there were a total of 16,042,306 deaths attributed to cardiovascular disease-related mortality in the USA, 8,034,209 (50.1%) of which occurred in urban areas (Supplemental Table 2). For all CVD-related deaths, 49.3% were in men and 92.6% were in those aged above 55. When stratified by race, 84.9% of deaths were in White individuals, 12.4% were in Black individuals, 2.2% were in Asians, and 0.5% were in American Indian individuals.

**Fig. 1:**
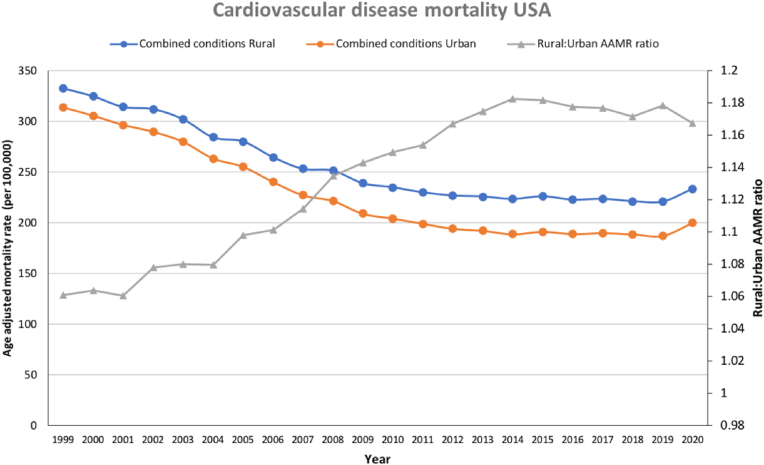
Age-adjusted mortality rate for CVD (all-cause mortality) in rural and urban areas, with rural-urban AAMR ratio provided as secondary axis.

**Fig. 2:**
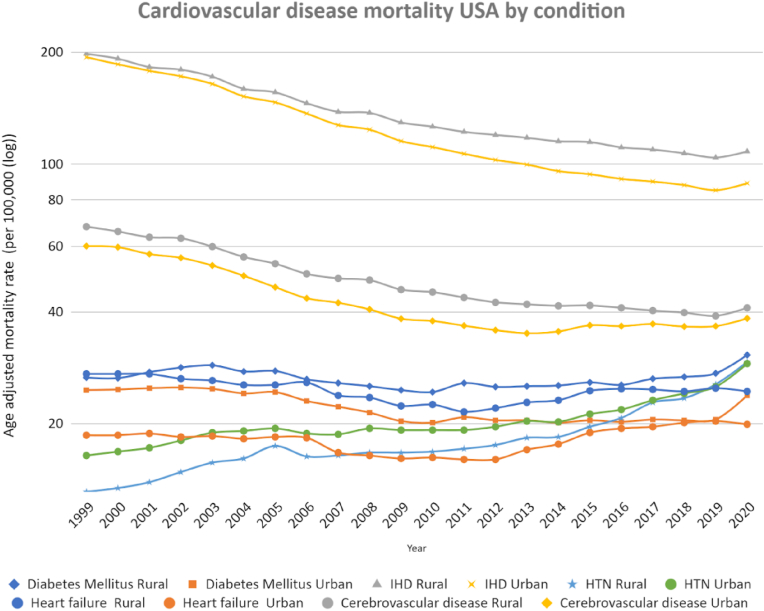
Age-adjusted mortality rate by disease subtypes and rural-urban designation (logarithmic scale).

Throughout the study period, AAMRs for overall CVD-related mortality were higher in rural than in urban areas for the whole study population as well as across age, sex, and racial groups (Table 1 and Fig. 1). AAMRs for individual contributors to CVD-related mortality were also higher in rural than urban areas throughout the study period, except for mortality related to hypertensive disease (Table 1 and Fig. 2). For all CVDs combined, AAMRs fell by −40.3% (AAPC = −2.7%) in urban areas and −33.6% (AAPC = −2.2%) in rural areas (p_trend_<0.001 for both) for the period 1999–2019 (Table 1). The rural-urban AAMR mortality gap increased from +6.0% in 1999 to +18.0% in 2019 (p_trend_<0.001); and subsequently was +17.0% in 2020. From 2019 to 2020, there was a 6.7% and 5.7% increase in AAMR in urban and rural areas, respectively. As a result of this reversal in previously improving CV mortality trends in rural and urban areas, in 2020, rural areas saw an approximate 10,093 (+6.4%) excess deaths whereas urban areas had 53,436 (+7.7%) excess deaths (Table 1).

**Table 1:** Age-adjusted mortality rate by demographic and condition, stratified into urban and rural areas.

	Urban (AAMR)								Rural (AAMR)						
		1999	2019	Δ% ‘99 to ‘19	AAPC (95% CI)	2020	Δ% ‘19 to ‘20	Excess deaths in 2020 (n,%)	1999	2019	Δ% ‘99 to ‘19	AAPC (95% CI)	2020	Δ% ‘19 to ‘20	Excess deaths in 2020 (n,%)
Combined		313.7 (313.0–314.5)	187.4 (186.9–187.8)	**−40.3**	**−2.7 (-3.1 to -2.3)**	200.0 (199.5–200.4)	**6.7**	53,436 (7.7%)	332.9 (331.4–334.5)	220.9 (219.7–222.0)	**−33.6**	**−2.2 (-2.6 to -1.9)**	233.5 (232.3–234.7)	**5.7**	10,093 (6.4%)
Demographics	Female	267.7 (266.8–268.6)	151.1 (150.5–151.6)	**−43.6**	**−3.2 (-3.6 to -2.9)**	160.5 (160.0–161.1)	**6.2**	27,728 (7.5%)	281.0 (279.2–282.9)	177.6 (176.2–178.9)	**−36.8**	**−2.5 (-2.8 to -2.2)**	187.2 (185.8–188.6)	**5.4**	23,489 (6.4%)
	Male	374.8 (373.5–376.2)	231.8 (231.0–232.6)	**−38.2**	**−2.6 (-3.0 to -2.2)**	247.6 (246.8–248.4)	**6.8**	24,282 (7.5%)	399.6 (396.9–402.4)	270.3 (268.3–272.2)	**−32.4**	**−2.1 (-2.4 to -1.7)**	286.3 (284.3–288.3)	**5.9**	20,997 (6.5%)
	Under 55	25.4 (25.2–25.6)	21.1 (20.9–21.3)	**−16.9**	**−1.1 (-1.3 to -0.9)**	24.4 (24.2–24.6)	**15.6**	7698 (15.9%)	29.5 (28.9–30.1)	31.8 (31.2–32.5)	7.8*	**0.3 (0.1 to 0.4)**	36.0 (35.3–36.6)	**13.2**	1339 (12.4%)
	55 and over	1375.0 (1371.7–1378.4)	799.3 (797.2–801.3)	**−41.9**	**−3.0 (-3.4 to -2.6)**	846.2 (844.2–848.3)	**5.9**	44,820 (6.9%)	1449.7 (1442.7–1456.7)	916.8 (911.9–921.7)	**−36.8**	**−2.5 (-2.8 to -2.1)**	960.7 (955.8–965.7)	**4.8**	8209 (5.6%)
	American Indian	212.6 (201.4–223.9)	115.9 (111.4–120.5)	**−45.5**	**−3.0 (-3.4 to -2.6)**	127.0 (122.4–131.6)	**9.6**	365 (11.4%)	372.1 (353.5–390.8)	218.9 (209.9–227.9)	**−41.2**	**−2.1 (-2.5 to -1.7)**	239.4 (230.2–248.7)	**9.4**	299 (10.9%)
	Asian	208.5 (204.5–212.5)	120.3 (118.8–121.9)	**−42.3**	**−2.7 (-3.1 to -2.3)**	133.8 (132.2–135.4)	**11.2**	3500 (12.6%)	298.5 (273.6–323.4)	137.0 (127.9–146.1)	**−54.1**	**−3.1 (-3.7 to -2.6)**	141.8 (132.8–150.8)	**3.5**	46 (4.7%)
	Black	420.1 (417.2–423.0)	254.6 (252.9–256.2)	**−39.4**	**−2.9 (-3.3 to -2.5)**	288.4 (286.7–290.2)	**13.3**	15,787 (14.3%)	440.2 (432.7–447.7)	297.8 (292.3–303.2)	**−32.4**	**−2.3 (-2.6 to -2.0)**	336.8 (331.0–342.5)	**13.1**	1934 (13.9%)
	White	304.9 (304.2–305.7)	182.6 (182.1–183.1)	**−40.1**	**−2.9 (-3.3 to -2.5)**	192.2 (191.7–192.7)	**5.3**	33,780 (6.1%)	325.5 (323.9–327.1)	215.4 (214.2–216.6)	**−33.8**	**−2.2 (-2.6 to -1.9)**	226.0 (224.8–227.2)	**4.9**	7861 (5.7%)
CVD conditions	DM	24.6 (24.4–24.9)	20.5 (20.4–20.7)	**−16.7**	**−1.3 (-1.6 to -1.0)**	23.8 (23.6–23.9)	**16.1**	13,224 (16.1%)	26.6 (26.2–27.1)	27.3 (26.9–27.7)	2.6*	**−0.3 (-0.6 to 0.0)**	30.6 (30.2–31.1)	**12.1**	2200 (11.0%)
	IHD	194.0 (193.4–194.5)	85.0 (84.6–85.3)	**−56.2**	**−4.4 (-4.7 to -4.1)**	88.8 (88.5–89.1)	**4.5**	23,192 (7.5%)	198.2 (196.9–199.4)	104.1 (103.3–104.9)	**−47.5**	**−3.3 (-3.5 to -3.0)**	108.1 (107.3–108.9)	**3.8**	4338 (6.0%)
	HTN	16.4 (16.2–16.6)	25.1 (24.9–25.3)	**53.1**	**1.7 (1.4 to 2.1)**	29.0 (28.8–29.2)	**15.5**	12,120 (12.1%)	13.1 (12.8–13.4)	25.5 (25.1–25.9)	**94.7**	**2.9 (2.4 to 3.4)**	29.3 (28.9–29.8)	**14.9**	1903 (9.7%)
	Heart failure	18.6 (18.4–18.8)	20.3 (20.1–20.4)	9.1*	**0.3 (-0.3 to 0.8)**	19.9 (19.8–20.1)	**−2.0**	−3406 (−4.9%)	27.2 (26.7–27.6)	24.9 (24.5–25.3)	**−8.5**^†^	**−0.7 (-1.1 to -0.3)**	24.4 (24.0–24.8)	**−2.0**	−590 (−3.6%)
	Cerebrovascular disease	60.1 (59.8–60.4)	36.6 (36.4–36.8)	**−39.1**	**−2.6 (-3.2 to -2.0)**	38.4 (38.2–38.6)	**4.9**	6805 (5.1%)	67.8 (67.1–68.5)	39.0 (38.5–39.5)	**−42.5**	**−2.9 (-3.2 to -2.6)**	41.0 (40.5–41.5)	**5.1**	1854 (6.7%)

All variables demonstrated statistical significance in AAMR trends with a p-value of <0.001, apart from those marked by the following: ‘*’ indicates p-value>0.05 and ‘†’ indicates p-value >0.001.

### 3.2 Cause specific cardiovascular death analysis

Changes in AAMR between 1999 and 2019 for individual components of overall CV mortality are shown in Table 1. There was significant temporal change in AAMR from 1999 to 2019 in ischemic heart disease (−56.2% (AAPC = −4.4%) in urban areas, −47.5% (AAPC = −3.3%) in rural areas), cerebrovascular disease (−39.1% (AAPC = −2.6%) in urban areas, −42.5% (AAPC = −2.9%) in rural areas), hypertension (+53.1% (AAPC = +1.7%) in urban areas, +94.7% (AAPC = +2.9%) in rural areas), diabetes in urban areas (−16.7%, AAPC = −1.3%), and heart failure in rural areas (−8.5%, AAPC = −0.7%); all p < 0.05. There was a non-significant temporal change observed in AAMR in diabetes-attributed mortality in rural areas and heart failure-attributed mortality in urban areas. The rural-urban difference in AAMR from 1999 to 2019 widened for Hypertension (+21.7%), ischemic heart disease (+20.3%), and diabetes (+25.0%) (p_trend_<0.001). The rural-urban difference significantly decreased from 46.2% to 22.6% for heart failure-related AAMR (p_trend_<0.001).

**Fig. 3:**
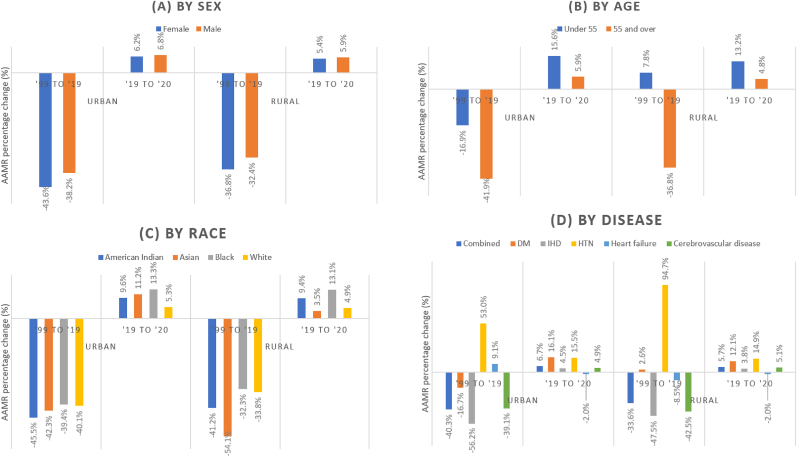
Percentage change in AAMR (1999–2019 & 2019–2020) across sub-groups.

From 2019 to 2020, there was a reversal of the decline in AMMR temporal trends for CV mortality in urban and rural areas (Fig. 3D). This was consistent for most individual causes of CV mortality, except for mortality from hypertension (which increased further by 15.5% in urban areas and 14.9% in rural areas), diabetes (which further increased by 12.1% in rural areas), and heart failure (which continued to demonstrate decreases in AAMR in urban and rural areas), see Fig. 3D. This translated to varying levels of excess mortality in 2020 (Table 1), with urban areas reporting more excess mortality than rural areas. The greatest individual CV contributor to excess mortality for 2020 was ischemic heart disease: 23,192 (+7.5%) in urban areas and 4338 (6.0%) in rural areas.

### 3.3 Sex analysis

In rural and urban areas, cardiovascular disease AAMRs were higher in males than females throughout the study period (Table 1). A decreasing temporal trend in CVD-related AAMRs were seen in both males and females to a greater degree in urban areas (Male AAPC = −2.6%, Female AAPC = −3.2%) than rural areas (Male AAPC = −2.1%, Female AAPC = −2.5%) from 1999 to 2019 (p_trend_<0.001). The male-female mortality gap (Male AAMR: Female AAMR ratio), from 1999 to 2019, increased in urban areas from a ratio of 1:1.4 to 1:1.5 and in rural areas from 1:1.4 to 1:1.5 (p_trend_<0.001).

In 2020, there was an increase in CVD-related AAMRs among males and females in both urban and rural areas (see Fig. 3A). As seen in Table 1, this translated to greater excess deaths in urban areas (Males 24,282 (7.5%), Females 27,728 (7.5%)) than rural areas (Males 20,997 (6.5%), Females 23,489 (6.4%)).

### 3.4 Age analysis

Between 1999 and 2019, amongst those aged under 55, there was a significant temporal increase in CVD-related AAMRs in rural areas (AAPC = +0.3%), whilst urban areas (AAPC = −1.1%) saw a significant decrease (both p_trend_<0.05, Table 1). In the same time period, those aged above 55 witnessed a temporal decline in CVD-related AAMRs of in both rural and urban areas, with AAPCs ranging from −2.5% to 3.0% (p_trend_<0.001), respectively. In the year 2020, there was an increase in CVD-related AAMRs across all age groups and rural-urban designation. The smallest change was a 4.8% increase in those greater than 55 in rural areas (urban equivalent: +5.9%), whereas the greatest change was a 15.6% increase in those under 55 in urban areas (rural equivalent: +13.2%) (see Fig. 3B). We estimate 44,820 (6.9%) excess deaths for those above 55 in urban areas and 8209 (5.6%) excess deaths in rural areas. For those below 55, there were 1339 (12.4%) excess deaths in rural areas and 7698 (15.9%) in urban areas (Table 1).

### 3.5 Race analysis

Black and American Indian patients had the highest cardiovascular AAMRs relative to White and Asian patients (see Supplementary Table 2). Each racial group saw higher AAMRs in rural areas. Between 1999 and 2019, across races and regardless of rural-urban designation, there was a statistically significant temporal decline in CVD-related AAMRs with AAPCs ranging from −2.1% in American Indians in rural areas to −3.1% in those of Asian origin in rural areas (p_trend_<0.001). 2020 saw a reversal of this trend, with the greatest relative magnitude in excess deaths seen in Black patients in urban (15,787 (14.3%)) and rural (1934 (13.9%)) areas (see Table 1).

## 4 Discussion

Our analysis of national CVD-related mortality between 1999 and 2020 with specific focus on differences between urban and rural regions demonstrates several important findings. Between 1999 and 2019 there was an overall decline in CVD-related mortality nationwide, which was more prominent in urban areas compared to rural areas. Significant declines in mortality were seen for most individual CVD causes in both urban and rural areas, except for hypertensive mortality which demonstrated an increase in both rural and urban areas. Reductions in CVD-related mortality between 1999 and 2019 were noted across sex, age, and all racial groups. Although CVD-related mortality increased in those under 55 years of age in rural areas. Mortality remained highest for Black Americans during the study period, both in rural and urban regions. The first year of the COVID pandemic in 2020 demonstrated reversal in prior decreases in CVD-related mortality across both rural and urban areas, with slightly greater increase in AAMRs in urban areas, leading to an estimated excess deaths of 10,093 and 53,436 respectively. Increases in CVD-related mortality in 2020 were noted for most individual causes of CVD mortality and across age, sex, and racial groups. These results have important implications for evaluating historical trends and the effects of the COVID pandemic on CVD-related mortality in urban and rural areas in the United States.

### 4.1 Overall mortality trends (and disease specific mortality trends)

The current study expands on prior literature regarding overall trends in CVD-related mortality between urban and rural areas prior to and during the first year of the COVID pandemic [1,4,6,12,[15], [16], [17], [18], [19]]. In recent decades and markedly from 2010 onwards, studies have noted a widening gap in CVD-related mortality between rural and urban areas over time; this has been attributed to lower socioeconomic status, older population, less access to healthcare, and reduced healthcare awareness in rural areas [[7], [8], [9]]. Novel findings in the current study include a breakdown of pre-COVID and early COVID mortality trends between urban and rural areas characterized by individual components of CVD-related mortality and other important demographic characteristics. There may be several reasons for the differential effects of the COVID pandemic on CVD outcomes in urban versus rural areas. Early COVID studies published in 2020 reported a greater disease burden in urban areas compared to rural areas [16,20,21], due to higher disease spread because of high population density, high degree of connectivity, crowded living conditions, inundated healthcare facilities and exposed occupations. These may explain the greater CVD-related percentage of excess mortality we report in urban areas in 2020 [[16], [17], [18],^22^]. It is also worth noting, these findings occurred at a time when vaccines were unavailable. Given the ongoing and evolving nature of the COVID pandemic, these findings highlight potential conditions requiring additional public health attention. Additionally, we identify a potential need for extra emphasis on the management of hypertensive diseases and diabetes in rural regions as increasing contributors to CVD mortality, and the effects of COVID on these conditions deserve further evaluation.

### 4.2 Trends by age

In this study, we also demonstrate important findings regarding the relationship between age and temporal trends in CVD-related mortality in urban and rural areas. While patients over 55 years of age derived significant reduction in CVD-related mortality (around 40%) in both urban and rural areas between 1999 and 2019, those under 55 years of age experienced a smaller 17% reduction in CVD mortality in urban areas and an increase in CVD mortality in rural areas. There may be several explanations for the finding of less prominent improvement in younger patients, particularly in rural areas. Smaller improvements or even increases in CVD-related mortality in younger patients have been described previously both in the US [[15], [23],^24^] and other industrialised countries [25,26]. Smaller improvements in CVD outcomes in younger patients have been attributed to lower uptake of treatments for CVD comorbidities including diabetes, hyperlipidemia, and heart failure, and the rise in CVD risk factors such as diabetes mellitus, obesity, and hypertension in younger populations [[27], [28], [29], [30], [31]]. Younger patients, particularly those in rural communities, may also have a greater burden of psychosocial comorbidities such as substance abuse and high risk behavior such as smoking and obesity, which may contribute to higher risk of CVD disease [32]. Additionally, younger patients in rural areas have been reported to have worse physical and mental health status, lower education status, as well as reduced access to care and lower amount of health insurance coverage [[33], [34], [35]]. Although we found that increases in CVD-related mortality between 2019 and 2020 were more prominent in urban versus rural areas for both adults under and over age 55, AAMRs remained significantly higher for both age groups in rural compared to urban areas. These findings highlight the need for additional efforts to improve rural health, with particular focus on CVD disease in younger Americans due to disparate effects on the pandemic on this cohort.

### 4.3 Racial trends

The current study also expands on prior literature regarding trends in CVD-related mortality based on race, with a focus on rural and urban disparities. Black patients experienced a greater decline in CVD-related mortality in urban areas compared to rural areas between 1999 and 2019, with similar increases in CVD-related mortality in urban and rural areas between 2019 and 2020. Black patients in rural areas have been reported to have higher rates of diabetes and hypertension compared to urban Black patients, as well as higher rates of uncontrolled diabetes and hypertension, which may be expected to translate into higher CVD-related mortality [36]. Members of underrepresented racial communities, including Black Americans have higher levels of socioeconomic deprivation, which is a by-product of systemic discriminatory policies that have limited access to education, occupations, and safe, affordable housing, all of which contribute directly to disparities in CVD-related mortality rates [35]. In addition to identifying differential mortality trends between Black Americans in urban versus rural areas, we also confirm findings of prior studies that CVD-related mortality in Black Americans remains higher than for other races. Mortality for White Americans, even in rural areas, remains lower than for Black Americans in either urban or rural areas throughout the study period. This also remained true for trends in mortality between 2019 and 2020, where we show that Black patients had greater increases in mortality in both rural and urban areas compared to White patients and other racial groups. Taken together, these findings highlight ongoing racial differences in care, which were further amplified by the first year of the COVID pandemic.

There are limitations to this study. On March 11, 2020, COVID-19 was declared by the World Health Organisation although the disease was prevalent from earlier in 2020. Due to concern for inaccurate diagnosis in the early phase of the pandemic, we did not evaluate ICD codes for COVID as a contributor to mortality. Excess CVD-related deaths in rural and urban regions in 2020 could have been a function of COVID-19 directly or due to indirect effects of COVID on the health delivery system. Moreover, the accuracy of coding for etiologies of mortality in 2020 would have been subject to regional biases as well as changes over time. Whether there are systemic differences in the accurate characterization of the cause of death between rural and urban areas cannot be determined and may affect the validity of the results. We were not able to adjust to baseline comorbidities, as well as other important potential confounders such as socioeconomic status, education, and occupation. Subgroup analysis with different age groups were not performed Additionally, as we used the 2013 US Census classification to determine rural-urban designation, we could not account for the change in the composition of rural and urban areas over the period of analysis. Rural areas may have experienced population growth and/or integration with an urban center since 2013. Moreover, we could not account for the possibility of those individuals who chose to migrate from rural-urban regions or vice versa.

## 5 Conclusion

In conclusion, we demonstrate that CVD-related disease mortality steadily improved from 1999 to 2019 in both rural and urban areas, although the improvements were greater in urban areas. Improvements in CVD-related mortality were similar in urban and rural areas among sexes, races, and patients over age 55, while those under age 55 experienced a decrease in CVD-related mortality in urban areas but an increase in CVD-related mortality in rural areas. Between 2019 and 2020, the first year of the COVID pandemic (when vaccines were unavailable), there was a reversal of previously improving CVD-related mortality trends in both urban and rural areas, with an increase in CVD-related mortality that was mildly more prominent in urban compared to rural areas. Increases in mortality in urban areas compared to rural areas in 2020 were similar based on sex, age, and racial groups and were seen for most individual components of CVD-related mortality. These results reinforce the need to focus on CVD care among both urban and rural communities; emergency medical services and planning has to be improved to be better capable of managing surges in healthcare demand during a natural disaster such as a pandemic. Decreased equitable access to good quality and timely healthcare leads to delayed care and worse patient outcomes.

## Disclosures

The authors have nothing to disclose.

## CRediT authorship contribution statement

**Saisunder S. Chaganty:** Writing – original draft, Literature Review, Formal analysis, Curation, Project administration. **Dmitry Abramov:** Writing – review & editing, Supervision, Methodology, Conceptualization. **Harriette G.C. Van Spall:** Writing – review & editing, Validation, Reviewing, Editing. **Renee P. Bullock-Palmer:** Writing – review & editing, Validation, Reviewing, Editing. **Vassilios Vassiliou:** Writing – review & editing, Validation, Reviewing, Editing. **Phyo Kyaw Myint:** Writing – review & editing, Validation, Reviewing, Editing. **Vijay Bang:** Writing – review & editing, Validation, Reviewing, Editing. **Ofer Kobo:** Writing – review & editing, Supervision, Methodology, Conceptualisation. **Mamas A. Mamas:** Writing – review & editing, Supervision, Methodology, Conceptualisation, Visualization.

## References

[1] Woolf S.H., Schoomaker H. (2019). Life expectancy and mortality rates in the United States, 1959-2017. JAMA.

[2] Iyer A.S., Cross S.H., Dransfield M.T., Warraich H.J. (2021). Urban-rural disparities in deaths from chronic lower respiratory disease in the United States. Am. J. Respir. Crit. Care Med..

[3] Vuong J.T. (2019). Mortality from heart failure and dementia in the United States: CDC WONDER 1999–2016. J. Card. Fail..

[4] Kobo O., Van Spall H.G.C., Mamas M.A. (2022). Urban-rural disparities in diabetes-related mortality in the USA 1999-2019. Diabetologia.

[5] Burton L.M., Lichter D.T., Baker R.S., Eason J.M. (2013). Inequality, family processes, and health in the “new” rural America. Am. Behav. Sci..

[6] Singh G.K., Siahpush M. (2014). Widening rural-urban disparities in life expectancy, U.S., 1969-2009. Am. J. Prev. Med..

[7] Richman L., Pearson J., Beasley C., Stanifer J. (2019). Addressing health inequalities in diverse, rural communities: an unmet need. SSM - Popul. Heal..

[8] Coughlin S.S. (2019). Continuing challenges in rural health in the United States. J. Environ. Heal. Sci..

[9] Long A.S., Hanlon A.L., Pellegrin K.L. (2018). Socioeconomic variables explain rural disparities in US mortality rates: implications for rural health research and policy. SSM - Popul. Heal..

[10] Sidney S. (2016). Recent trends in cardiovascular mortality in the United States and public health goals. JAMA Cardiol.

[11] Shah N.S. (2019). Trends in cardiometabolic mortality in the United States, 1999-2017. JAMA.

[12] Cross S.H. (2020). Rural-urban differences in cardiovascular mortality in the US, 1999-2017. JAMA.

[13] World Health Organisation (2021).

[14] Ingram D.D., Franco S.J. (2013). NCHS urban-rural classification scheme for counties. Vital Health Stat.

[15] Kobo O. (2022). Has the first year of the COVID-19 pandemic reversed the trends in CV mortality between 1999-2019 in the United States?. Eur. Hear. journal. Qual. care Clin. outcomes.

[16] Cuadros D.F., Branscum A.J., Mukandavire Z., Miller F.D., MacKinnon N. (2021). Dynamics of the COVID-19 epidemic in urban and rural areas in the United States. Ann. Epidemiol..

[17] Hamidi S., Ewing R., Sabouri S. (2020). Longitudinal analyses of the relationship between development density and the COVID-19 morbidity and mortality rates: early evidence from 1,165 metropolitan counties in the United States. Health Place.

[18] Kim B. (2021). COVID-19 testing, case, and death rates and spatial socio-demographics in New York City: an ecological analysis as of June 2020. Health Place.

[19] Kobo O. (2022). CKD-associated cardiovascular mortality in the United States: temporal trends from 1999 to 2020. Kidney Med.

[20] Wang D., Chonody J.M., Krase K., Luzuriaga L. (2021). Coping with and adapting to COVID-19 in rural United States and Canada. Fam. Soc..

[21] Sharifi A., Khavarian-Garmsir A.R. (2020). The COVID-19 pandemic: impacts on cities and major lessons for urban planning, design, and management. Sci. Total Environ..

[22] Frumkin H. (2021). COVID-19, the built environment, and health. Environ. Health Perspect..

[23] Oseran A.S., Afari M.E., Barrett C.D., Lewis G.D., Thomas S.S. (2021). Beyond the stethoscope: managing ambulatory heart failure during the COVID-19 pandemic. ESC Hear. Fail..

[24] Jain V. (2022). Demographic and regional trends of heart failure-related mortality in young adults in the US, 1999-2019. JAMA Cardiol.

[25] Sekikawa A. (2015). Continuous decline in mortality from coronary heart disease in Japan despite a continuous and marked rise in total cholesterol: Japanese experience after the Seven Countries Study. Int. J. Epidemiol..

[26] Allender S., Scarborough P., O'Flaherty M., Capewell S. (2008). Patterns of coronary heart disease mortality over the 20th century in England and Wales: possible plateaus in the rate of decline. BMC Publ. Health.

[27] Andes L.J., Cheng Y.J., Rolka D.B., Gregg E.W., Imperatore G. (2020). Prevalence of prediabetes among adolescents and young adults in the United States, 2005-2016. JAMA Pediatr..

[28] Ogden C.L. (2016). Trends in obesity prevalence among children and adolescents in the United States, 1988-1994 through 2013-2014. JAMA.

[29] Andersson C., Vasan R.S. (2018). Epidemiology of cardiovascular disease in young individuals. Nat. Rev. Cardiol..

[30] D., M. E. & D., C. A. Coronary (2019). Artery disease in young adults. J. Am. Coll. Cardiol..

[31] Khan S.U. (2020). Trends in cardiovascular deaths among young adults in the United States, 1999 to 2018. Am. J. Cardiol..

[32] Kochanek K.D., Arias E., Bastian B.A. (2016). The effect of changes in selected age-specific causes of death on non-hispanic white life expectancy between 2000 and 2014. NCHS Data Brief.

[33] West A., Weeks W.B. (2006). Physical and mental health and access to care among nonmetropolitan Veterans Health Administration patients younger than 65 years. J. Rural Heal. Off. J. Am. Rural Heal. Assoc. Natl. Rural Heal. Care Assoc..

[34] Eberhardt M.S., Pamuk E.R. (2004). The importance of place of residence: examining health in rural and nonrural areas. Am. J. Publ. Health.

[35] Harrington R.A. (2020). Call to action: rural health: a presidential advisory from the American heart association and American stroke association. Circulation.

[36] Mainous A.G., King D.E., Garr D.R., Pearson W.S. (2004). Race, rural residence, and control of diabetes and hypertension. Ann. Fam. Med..

