# Supplementary material for: Rural and urban disparities in cardiovascular disease-related mortality in the USA over 20 years; have the trends been reversed by COVID-19?
Source: Int J Cardiol Cardiovasc Risk Prev. 2023 Aug 30;19:200202. doi: 10.1016/j.ijcrp.2023.200202 (PMC10477062; doi:10.1016/j.ijcrp.2023.200202)
Supplement: Multimedia component 1 [file mmc1.docx]

| **ICD-10 Codes** | **Diseases** |
| --- | --- |
| E10-E14 | Diabetes mellitus |
| I10-I15 | Hypertensive diseases |
| I20-I25 | Ischaemic heart diseases |
| I50.0, I50.1, I50.9 | Heart failure |
| I60-I69 | Cerebrovascular diseases |

Supplemental table 1: List of ICD-10 codes used and classed as ‘cardiovascular disease-related’ conditions

|  |  | **Urban (total no. of deaths)** | | | | | **Rural (total no. of deaths)** | | | | |
| --- | --- | --- | --- | --- | --- | --- | --- | --- | --- | --- | --- |
|  |  | 1999 | 2019 | **% Change '99 to '19** | 2020 | **% Change '19 to '20** | 1999 | 2019 | **% Change '99 to '19** | 2020 | **% Change '19 to '20** |
| Combined | | 690125 | 639827 | **-7.3%** | 694619 | **8.5 %** | 173209 | 146974 | **-15.2%** | 156505 | **6.5%** |
| Demographic | Female | 370421 | 302395 | **-18.4%** | 325457 | **7.6%** | 92061 | 67631 | **-26.5%** | 71291 | **5.4%** |
|  | Male | 319704 | 337432 | **5.6%** | 369162 | **9.4%** | 81148 | 79343 | **-2.2%** | 85214 | **7.4%** |
|  | Under 55 | 46082 | 42343 | **-8.1%** | 48503 | **14.6%** | 10048 | 9646 | **-4.0%** | 10778 | **11.7%** |
|  | 55 and over | 643997 | 597448 | **-7.2%** | 646098 | **8.1%** | 163161 | 137325 | **-15.8%** | 145724 | **6.1%** |
|  | American Indian | 1605 | 2778 | **73.1%** | 3210 | **15.6%** | 1681 | 2405 | **43.1%** | 2739 | **13.9%** |
|  | Asian | 11711 | 23801 | **103.2%** | 27836 | **17.0%** | 579 | 898 | **55.1%** | 978 | **8.9%** |
|  | Black | 84847 | 94192 | **11.0%** | 110365 | **17.2%** | 13308 | 12097 | **-9.1%** | 13922 | **15.1%** |
|  | White | 591962 | 519056 | **-12.3%** | 553208 | **6.6%** | 157641 | 131574 | **-16.5%** | 138866 | **5.5%** |
| CVD condition | DM | 54747 | 69848 | **27.6%** | 82149 | **17.6%** | 13652 | 17799 | **30.4%** | 20039 | **12.6%** |
|  | IHD | 426797 | 291354 | **-31.7%** | 310052 | **6.4%** | 102862 | 69546 | **-32.4%** | 72768 | **4.6%** |
|  | HTN | 36171 | 85339 | **135.9%** | 100467 | **17.7%** | 6826 | 16733 | **145.1%** | 19530 | **16.7%** |
|  | Heart failure* | 40542 | 69331 | **71.0%** | 69246 | **-0.1%** | 14371 | 16846 | **17.2%** | 16609 | **-1.4%** |
|  | Cerebrovascular disease | 131868 | 123955 | **-6.0%** | 132705 | **7.1%** | 35498 | 26050 | **-26.6%** | 27559 | **5.8%** |

Supplemental table 2: Number of deaths by demographic and condition. Please note: for a certain number of patients (1999- n=46 in urban areas, 2019- n=36 in urban areas and n=3 in rural areas, 2020- n=18 in urban areas and n=3 in rural areas) the age at death was not reported, hence the ‘under 55’ and ‘55 and over’ do not sum to the combined total.

|  | Urban (AAMR) | | | | Rural (AAMR) | | | |
| --- | --- | --- | --- | --- | --- | --- | --- | --- |
|  | 2010 AAMR | 2019 AAMR | Δ% AAMR from 2010 to 2019 | AAPC (95% CI) | 2010 AAMR | 2019 AAMR | Δ% AAMR from 2010 to 2019 | AAPC (95% CI) |
| Combined | 204.4 | 187.4 | -8.3% | -0.90 (-1.20 to -0.60) | 235 | 220.9 | -6.0% | -0.70 (-1.00 to -0.30) |
| Female | 169.6 | 151.1 | -10.9% | -1.20 (-1.60 to -0.80) | 194.6 | 177.6 | -8.7% | -0.90 (-1.10 to -0.70) |
| Male | 248.6 | 231.8 | -6.8% | -0.60 (-0.90 to -0.40) | 283.3 | 270.3 | -4.6% | -0.50 (-0.80 to -0.10) |
| Under 55 | 21.8 | 21.1 | -3.2% | -0.20 (-0.40 to -0.10) | 29.6 | 31.8 | 7.4% | 0.70 (0.30 to 1.10) |
| 55 and over | 876.6 | 799.3 | -8.8% | -1.00 (-1.30 to -0.60) | 991.2 | 916.8 | -7.5% | -0.80 (-1.20 to -0.50) |
| American Indian | 142 | 115.9 | -18.4% | -1.60 (-2.30 to -1.00) | 247.2 | 218.9 | -11.4% | -1.40 (-2.20 to -0.60) |
| Asian | 137.9 | 120.3 | -12.8% | -1.20 (-1.90 to -0.40) | 148 | 137 | -7.4% | -1.10 (-1.90 to -0.40) |
| Black | 281 | 254.6 | -9.4% | -0.90 (-1.30 to -0.60) | 320.5 | 297.8 | -7.1% | -0.70 (-1.10 to -0.30) |
| White | 198.1 | 182.6 | -7.8% | -0.80 (-1.20 to -0.50) | 229.3 | 215.4 | -6.1% | -0.70 (-1.00 to -0.30) |
| DM | 20.1 | 20.5 | 2.0% | 0.00 (-0.30 to 0.30) | 24.3 | 27.3 | 12.3% | 1.00 (0.50 to 1.40) |
| IHD | 111.1 | 85 | -23.5% | -2.80 (-3.10 to -2.60) | 126.1 | 104.1 | -17.4% | -2.00 (-2.10 to -1.80) |
| HTN | 19.2 | 25.1 | 30.7% | 3.10 (2.60 to 3.60) | 16.8 | 25.5 | 51.8% | 4.70 (3.80 to 5.60) |
| Heart failure | 16.2 | 20.3 | 25.3% | 3.10 (2.50 to 3.80) | 22.5 | 24.9 | 10.7% | 1.60 (1.00 to 2.30) |
| Cerebrovascular disease | 37.8 | 36.6 | -3.2% | -0.20 (-1.30 to 0.80) | 45.2 | 39 | -13.7% | -1.50 (-2.00 to -1.10) |

Supplemental table 3: Projected excess deaths based on the 2010 to 2019 trend. (AAPC - Average Annual Percent Change, AAMR – Age adjusted mortality rate per 100,000)

| **Year** | **All cause-mortality age-adjusted mortality ratio (AAMR)** | | **Rural-urban gap** |
| --- | --- | --- | --- |
|  | **Rural** | **Urban** |  |
| 1999 | 332.9 | 313.7 | 1.061 |
| 2000 | 325 | 305.5 | 1.064 |
| 2001 | 314.5 | 296.5 | 1.061 |
| 2002 | 312.3 | 289.7 | 1.078 |
| 2003 | 302.3 | 279.9 | 1.080 |
| 2004 | 284.5 | 263.5 | 1.080 |
| 2005 | 280.4 | 255.3 | 1.098 |
| 2006 | 264.8 | 240.4 | 1.101 |
| 2007 | 253.3 | 227.3 | 1.114 |
| 2008 | 251.5 | 221.6 | 1.135 |
| 2009 | 239 | 209.1 | 1.143 |
| 2010 | 235 | 204.4 | 1.150 |
| 2011 | 230 | 199.3 | 1.154 |
| 2012 | 226.9 | 194.4 | 1.167 |
| 2013 | 225.9 | 192.3 | 1.175 |
| 2014 | 223.5 | 189 | 1.183 |
| 2015 | 226.1 | 191.3 | 1.182 |
| 2016 | 222.8 | 189.2 | 1.178 |
| 2017 | 223.6 | 190 | 1.177 |
| 2018 | 221.3 | 188.9 | 1.172 |
| 2019 | 220.9 | 187.4 | 1.179 |
| 2020 | 233.5 | 200 | 1.167 |

Supplemental table 4: Age adjusted mortality by year in rural and urban areas, including rural-urban AAMR ratio. This table has been used to create Figure 1

|  | **Urban (AAMR)** | | | | | | | | **Rural (AAMR)** | | | | | | | |
| --- | --- | --- | --- | --- | --- | --- | --- | --- | --- | --- | --- | --- | --- | --- | --- | --- |
|  |  | 1999 | 2019 | Δ% '99 to '19 | AAPC (95% CI) | 2020 | Δ% '19 to '20 | Excess deaths in 2020 (n,%) | 1999 | 2019 | Δ% '99 to '19 | AAPC (95% CI) | 2020 | Δ% '19 to '20 | Excess deaths in 2020 (n,%) |  |
| Combined | | 313.7 (313.0-314.5) | 187.4 (186.9-187.8) | **-40.3** | **-2.7 (-3.1 to -2.3)** | 200.0 (199.5-200.4) | **6.7** | 53436 (7.7%) | 332.9 (331.4-334.5) | 220.9 (219.7-222.0) | **-33.6** | **-2.2 (-2.6 to -1.9)** | 233.5 (232.3-234.7) | **5.7** | 10093 (6.4%) |  |
| Demographics | Female | 267.7 (266.8-268.6) | 151.1 (150.5-151.6) | **-43.6** | **-3.2 (-3.6 to -2.9)** | 160.5 (160.0-161.1) | **6.2** | 27728 (7.5%) | 281.0 (279.2-282.9) | 177.6 (176.2-178.9) | **-36.8** | **-2.5 (-2.8 to -2.2)** | 187.2 (185.8-188.6) | **5.4** | 23489 (6.4%) |  |
|  | Male | 374.8 (373.5-376.2) | 231.8 (231.0-232.6) | **-38.2** | **-2.6 (-3.0 to -2.2)** | 247.6 (246.8-248.4) | **6.8** | 24282 (7.5%) | 399.6 (396.9-402.4) | 270.3 (268.3-272.2) | **-32.4** | **-2.1 (-2.4 to -1.7)** | 286.3 (284.3-288.3) | **5.9** | 20997 (6.5%) |  |
|  | Under 55 | 25.4 (25.2-25.6) | 21.1 (20.9-21.3) | **-16.9** | **-1.1 (-1.3 to -0.9)** | 24.4 (24.2-24.6) | **15.6** | 7698 (15.9%) | 29.5 (28.9-30.1) | 31.8 (31.2-32.5) | **7.8*** | **0.3 (0.1 to 0.4)** | 36.0 (35.3-36.6) | **13.2** | 1339 (12.4%) |  |
|  | 55 and over | 1,375.0 (1,371.7-1,378.4) | 799.3 (797.2-801.3) | **-41.9** | **-3.0 (-3.4 to -2.6)** | 846.2 (844.2-848.3) | **5.9** | 44820 (6.9%) | 1,449.7 (1,442.7-1,456.7) | 916.8 (911.9-921.7) | **-36.8** | **-2.5 (-2.8 to -2.1)** | 960.7 (955.8-965.7) | **4.8** | 8209 (5.6%) |  |
|  | American Indian | 212.6 (201.4-223.9) | 115.9 (111.4-120.5) | **-45.5** | **-3.0 (-3.4 to -2.6)** | 127.0 (122.4-131.6) | **9.6** | 365 (11.4%) | 372.1 (353.5-390.8) | 218.9 (209.9-227.9) | **-41.2** | **-2.1 (-2.5 to -1.7)** | 239.4 (230.2-248.7) | **9.4** | 299 (10.9%) |  |
|  | Asian | 208.5 (204.5-212.5) | 120.3 (118.8-121.9) | **-42.3** | **-2.7 (-3.1 to -2.3)** | 133.8 (132.2-135.4) | **11.2** | 3500 (12.6%) | 298.5 (273.6-323.4) | 137.0 (127.9-146.1) | **-54.1** | **-3.1 (-3.7 to -2.6)** | 141.8 (132.8-150.8) | **3.5** | 46 (4.7%) |  |
|  | Black | 420.1 (417.2-423.0) | 254.6 (252.9-256.2) | **-39.4** | **-2.9 (-3.3 to -2.5)** | 288.4 (286.7-290.2) | **13.3** | 15787 (14.3%) | 440.2 (432.7-447.7) | 297.8 (292.3-303.2) | **-32.4** | **-2.3 (-2.6 to -2.0)** | 336.8 (331.0-342.5) | **13.1** | 1934 (13.9%) |  |
|  | White | 304.9 (304.2-305.7) | 182.6 (182.1-183.1) | **-40.1** | **-2.9 (-3.3 to -2.5)** | 192.2 (191.7-192.7) | **5.3** | 33780 (6.1%) | 325.5 (323.9-327.1) | 215.4 (214.2-216.6) | **-33.8** | **-2.2 (-2.6 to -1.9)** | 226.0 (224.8-227.2) | **4.9** | 7861 (5.7%) |  |
| CVD conditions | DM | 24.6 (24.4-24.9) | 20.5 (20.4-20.7) | **-16.7** | **-1.3 (-1.6 to -1.0)** | 23.8 (23.6-23.9) | **16.1** | 13224 (16.1%) | 26.6 (26.2-27.1) | 27.3 (26.9-27.7) | **2.6*** | **-0.3 (-0.6 to 0.0)** | 30.6 (30.2-31.1) | **12.1** | 2200 (11.0%) |  |
|  | IHD | 194.0 (193.4-194.5) | 85.0 (84.6-85.3) | **-56.2** | **-4.4 (-4.7 to -4.1)** | 88.8 (88.5-89.1) | **4.5** | 23192 (7.5%) | 198.2 (196.9-199.4) | 104.1 (103.3-104.9) | **-47.5** | **-3.3 (-3.5 to -3.0)** | 108.1 (107.3-108.9) | **3.8** | 4338 (6.0%) |  |
|  | HTN | 16.4 (16.2-16.6) | 25.1 (24.9-25.3) | **53.1** | **1.7 (1.4 to 2.1)** | 29.0 (28.8-29.2) | **15.5** | 12120 (12.1%) | 13.1 (12.8-13.4) | 25.5 (25.1-25.9) | **94.7** | **2.9 (2.4 to 3.4)** | 29.3 (28.9-29.8) | **14.9** | 1903 (9.7%) |  |
|  | Heart failure | 18.6 (18.4-18.8) | 20.3 (20.1-20.4) | **9.1*** | **0.3 (-0.3 to 0.8)** | 19.9 (19.8-20.1) | **-2.0** | -3406 (-4.9%) | 27.2 (26.7-27.6) | 24.9 (24.5-25.3) | **-8.5**^†^ | **-0.7 (-1.1 to -0.3)** | 24.4 (24.0-24.8) | **-2.0** | -590 (-3.6%) |  |
|  | Cerebrovascular disease | 60.1 (59.8-60.4) | 36.6 (36.4-36.8) | **-39.1** | **-2.6 (-3.2 to -2.0)** | 38.4 (38.2-38.6) | **4.9** | 6805 (5.1%) | 67.8 (67.1-68.5) | 39.0 (38.5-39.5) | **-42.5** | **-2.9 (-3.2 to -2.6)** | 41.0 (40.5-41.5) | **5.1** | 1854 (6.7%) |  |

Table 1: Age-adjusted mortality rate by demographic and condition, stratified into urban and rural areas. All variables demonstrated statistical significance in AAMR trends with a p-value of <0.001, apart from those marked by the following: ‘*’ indicates p-value>0.05 and ‘†’ indicates p-value >0.001.


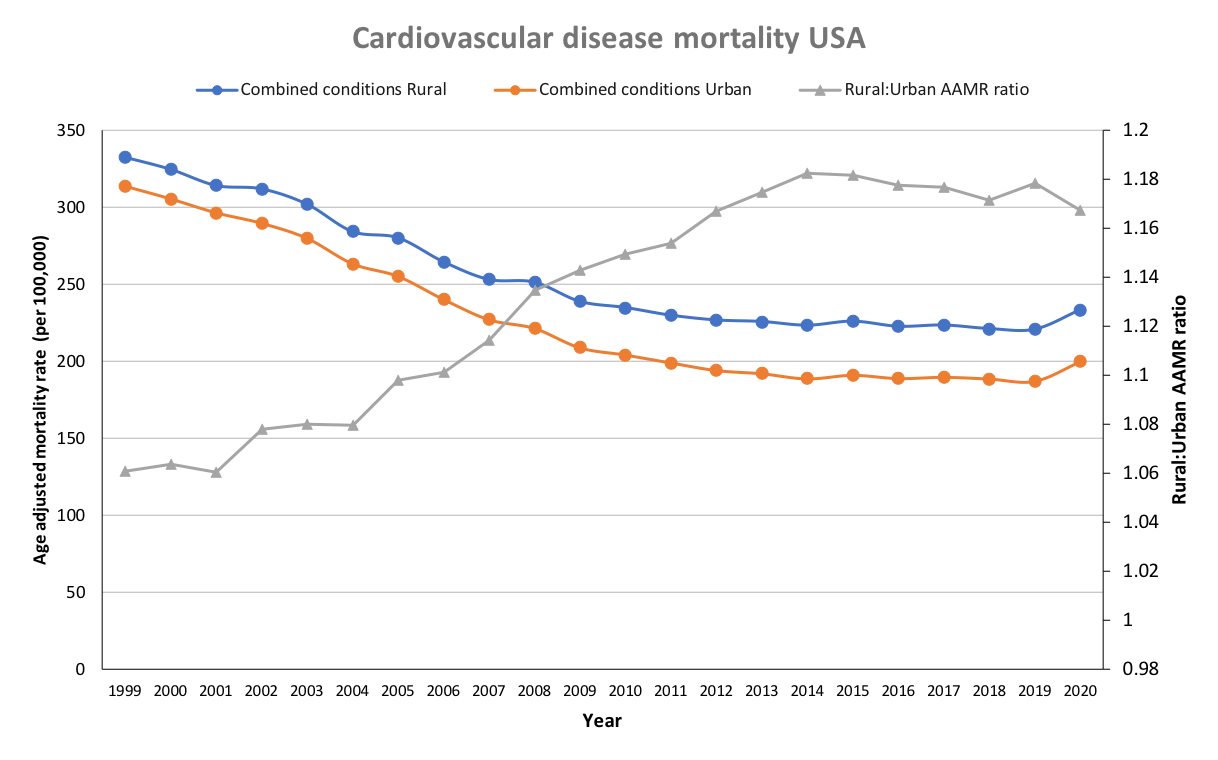
Figure1: Age-adjusted mortality rate for CVD (all-cause mortality) in rural and urban areas, with rural-urban AAMR ratio provided as secondary axis


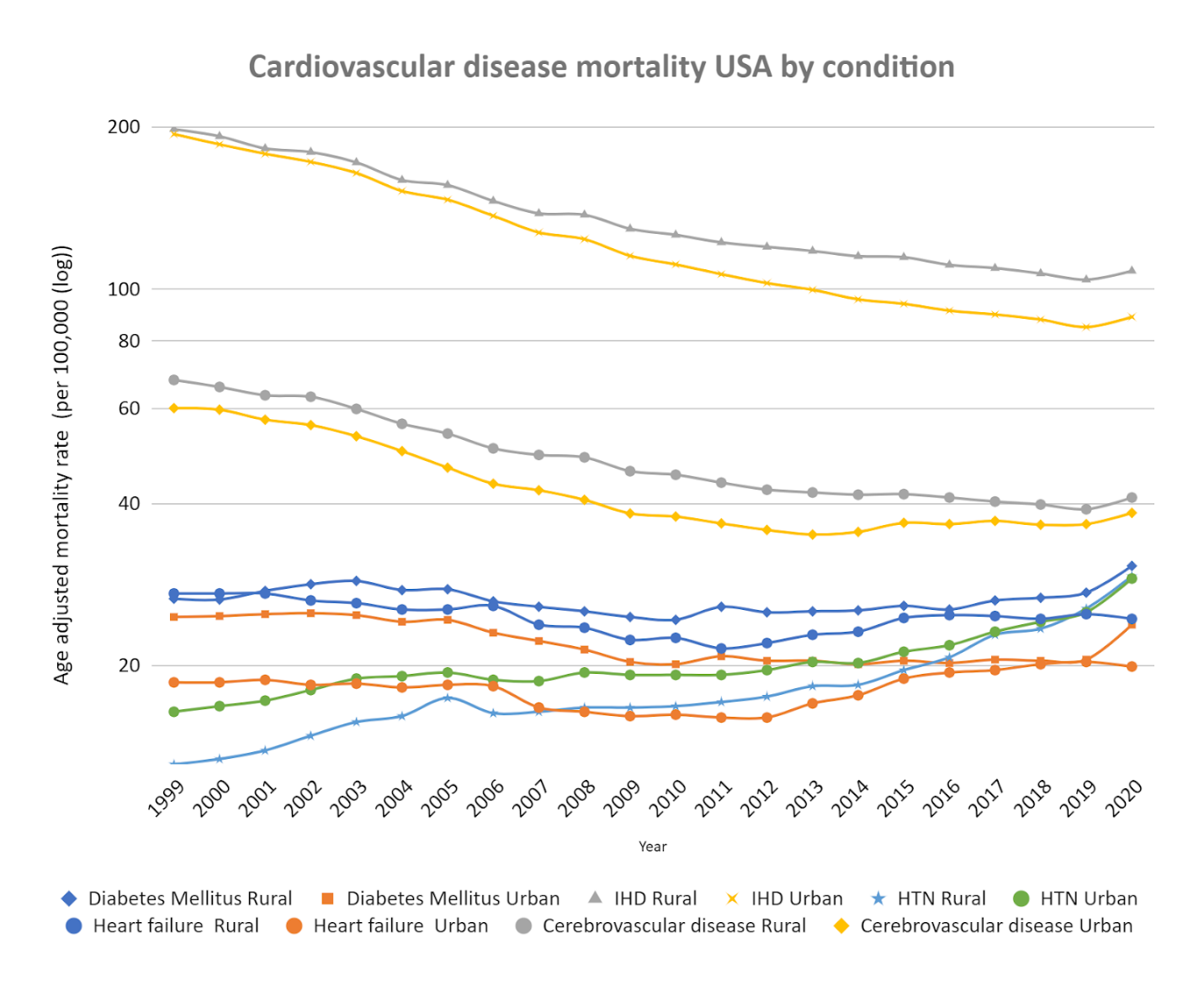


Figure 2: Age-adjusted mortality rate by disease subtypes and rural-urban designation (logarithmic scale)


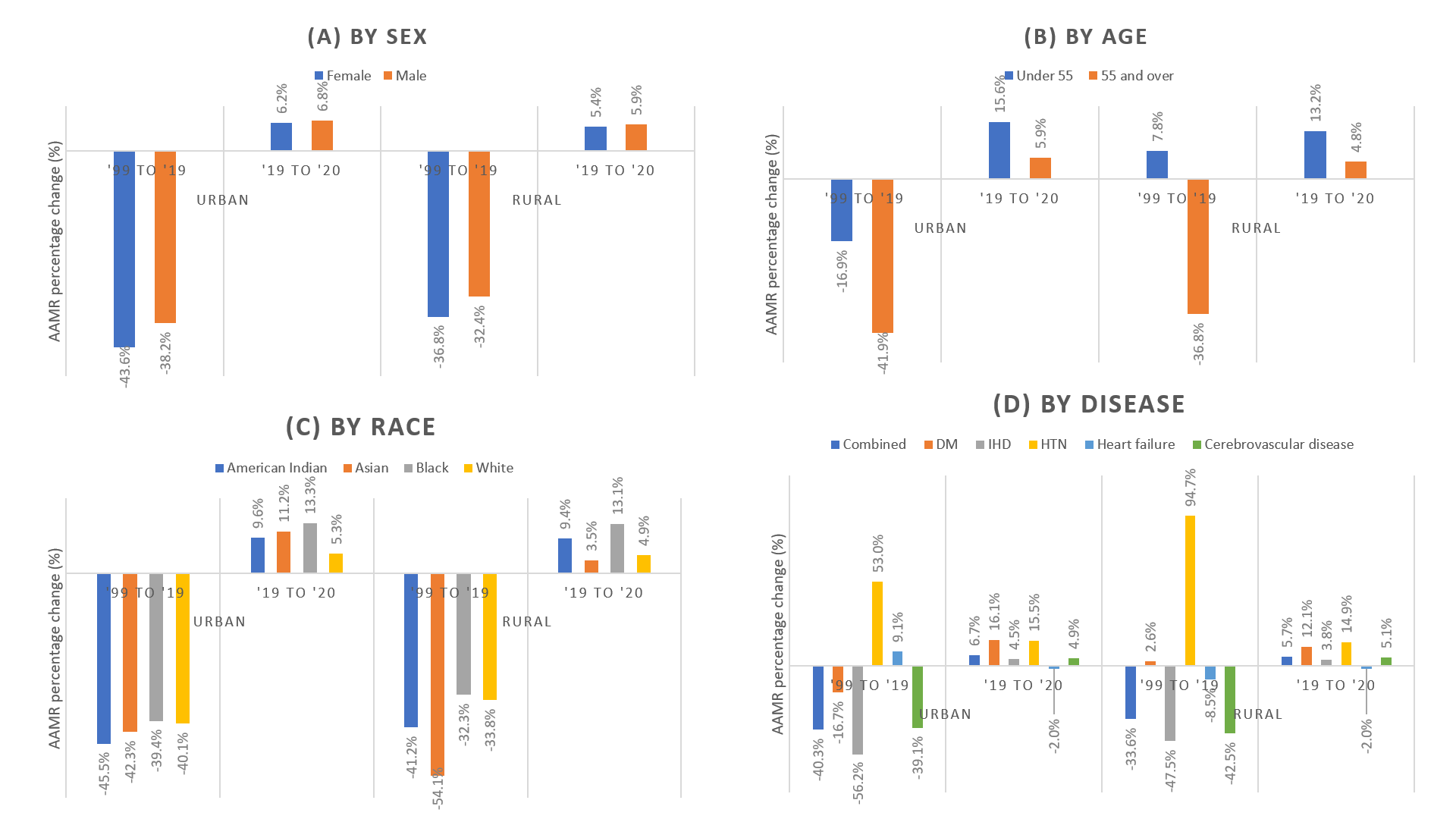
Figure 3: Percentage change in AAMR (1999-2019 & 2019-2020) across sub-groups
